# Identification and verification of circRNA biomarkers for coronary artery disease based on WGCNA and the LASSO algorithm

**DOI:** 10.1186/s12872-024-03972-2

**Published:** 2024-06-17

**Authors:** Qilong Zhong, Shaoyue Jin, Zebo Zhang, Haiyan Qian, Yanqing Xie, Peiling Yan, Wenming He, Lina Zhang

**Affiliations:** 1 General Practice Department, The Seventh Hospital of Ningbo, Ningbo, Zhejiang China; 2 Zhejiang Key Laboratory of Pathophysiology, Health Science Center, Ningbo University, Ningbo, Zhejiang China; 3 School of Public Health, Health Science Center, Ningbo University, Ningbo, Zhejiang China; 4 Institute of Geriatrics, The First Affiliated Hospital of Ningbo University, Ningbo, Zhejiang China; 5 Ningbo Municipal Center for Disease Control and Prevention, Ningbo, China

**Keywords:** Coronary artery disease, Circular RNA, Biomarker, WGCNA, LASSO

## Abstract

**Background:**

The role of circular RNAs (circRNAs) as biomarkers of coronary artery disease (CAD) remains poorly explored. This study aimed to identify and validate potential circulating circRNAs as biomarkers for the diagnosis of CAD.

**Methods:**

The expression profile of circRNAs associated with CAD was obtained from Gene Expression Omnibus (GEO) database. Differential expression analysis, weighted gene co-expression network analysis (WGCNA) and least absolute shrinkage and selection operation (LASSO) were employed to identify CAD-related hub circRNAs. The expression levels of these hub circRNAs were validated using qRT-PCR in blood samples from 100 CAD patients and 100 controls. The diagnostic performance of these circRNAs was evaluated through logistic regression analysis, receiver operator characteristic (ROC) analysis, integrated discrimination improvement (IDI), and net reclassification improvement (NRI). Functional enrichment analyses were performed to predict the possible mechanisms of circRNAs in CAD.

**Results:**

A total of ten CAD-related hub circRNAs were identified through WGCNA and LASSO analysis. Among them, hsa_circ_0069972 and hsa_circ_0021509 were highly expressed in blood samples of CAD patients, and they were identified as independent predictors after adjustment for relevant confounders. The area under the ROC curve for hsa_circ_0069972 and hsa_circ_0021509 was 0.760 and 0.717, respectively. The classification of patients was improved with the incorporation of circRNAs into the clinical model composed of conventional cardiovascular risk factors, showing an IDI of 0.131 and NRI of 0.170 for hsa_circ_0069972, and an IDI of 0.111 and NRI of 0.150 for hsa_circ_0021509. Functional enrichment analyses revealed that the hsa_circ_0069972-miRNA-mRNA network was enriched in *TGF-β*、*FoxO* and *Hippo* signaling pathways, while the hsa_circ_0021509-miRNA-mRNA network was enriched in *PI3K/Akt* and *MAPK* signaling pathways.

**Conclusion:**

Hsa_circ_0069972 and hsa_circ_0021509 were identified by integrated analysis, and they are highly expressed in CAD patients. They may serve as novel biomarkers for CAD.

**Supplementary Information:**

The online version contains supplementary material available at 10.1186/s12872-024-03972-2.

## Introduction

Coronary artery disease (CAD), which is known as a silent and progressive chronic disease, has become a major global health problem [1]. Lifestyle modifications, pharmacotherapy and coronary revascularisation have facilitated the clinical management of patients with symptoms suggestive of CAD [2, 3]. In current clinical practice, invasive coronary angiography remains the gold standard method for diagnosing CAD. However, for those with inconclusive imaging and high clinical likelihood, approximately 30–70% of patients with signs and/or symptoms of ischemia have non-obstructive coronary arteries [4]. Consequently, there is a necessity to identify non-invasive biomarkers for CAD to provide complementary diagnostic information and reduce unnecessary invasive procedures.

Various protein- or gene-based blood biomarkers associated with an increased risk for CAD have been identified. However, few have yet been shown to have a diagnostic impact that would affect patient management of CAD [5]. In this context, circular RNAs (circRNAs), a class of non-coding RNAs, have been proposed as potential diagnostic or prognostic biomarkers for the management of CAD patients [6]. One of the physiological roles of circRNAs is to function as miroRNA (miRNA) sponges through their binding sites, which modulates miRNA activity, thereby regulating the target genes of miRNAs [7]. Increasing evidence suggests that misregulation of circRNAs is associated with human diseases, such as cancer [8], cardiovascular disease [9] and neurological disorders [10]. CircRNAs are generated by back-splicing of precursor mRNA and have a circular covalently closed structure, which endowing circRNAs with a higher tolerance to exonuclease digestion and a longer half-time than linear RNAs [11]. Moreover, circRNAs can be specifically and differentially expressed not only in tissues but also in fluids [12], and their expression is dynamically regulated under various pathologic conditions of CAD [13]. A recent study showed that there are differences in circRNA expression profiles in peripheral blood between CAD patients and controls, suggesting that circRNAs may play a significant role in the development of CAD [14]. However, the role of circRNAs as biomarkers of CAD remains poorly explored.

In the present study, we aimed to identify and validate potential circulating circRNAs as biomarkers for the diagnosis of CAD. We used a CAD microarray dataset obtained from the GEO database. Weighted gene co-expression network analysis (WGCNA) and least absolute shrinkage and selection operator (LASSO) regression analysis were conducted to identify CAD-related hub circRNAs, and then quantitative real-time PCR (qRT-PCR) was performed to validate the expression of these circRNAs in the peripheral blood of CAD patients. The values of the hub circRNAs were evaluated as potential biomarkers for CAD. Moreover, circRNA-miRNA-mRNA networks were constructed and functional enrichment analyses were performed to predict the potential mechanisms and functions of circRNAs in CAD.

## Materials and methods

### Study population

In this study, we continuously enrolled 100 CAD patients and 100 age- and gender- matched non-CAD controls between September 2021 to March 2023, from the Departments of Cardiovascularology Medicine and Emergency Medicine, the First Affiliated Hospital of Ningbo University. All participants underwent coronary artery angiography during the hospitalization without prior percutaneous coronary intervention (PCI) or coronary artery bypass graft (CABG) surgery. The diagnosis of CAD was based on the percentage narrowing of each coronary artery segment, with at least one major coronary artery (the left main coronary trunk, anterior descending branch, circumflex artery, and right coronary artery) stenosis ≥ 50%. Exclusion criteria encompassed congenital heart disease, rheumatic valvular disease, myocardiopathy, severe heart failure, stroke, malignant neoplasms, acute or chronic infectious diseases, autoimmune diseases, severe liver or kidney dysfunction, and mental disorders. Following overnight fasting, blood samples were collected from all participants using EDTA anticoagulated tubes via venipuncture. In addition, detailed clinical and demographic data were collected for each participant on standardized forms.

This study was approved by the Ethics Committee of Health Science Center of Ningbo University (NBU-2020-114), and informed consent was obtained from all participants or their families.

### Data collection and differential expression analysis

The CAD-related circRNA expression microarray dataset GSE115733 [14], comprising 24 CAD patients and 7 controls, was obtained from the NCBI GEO database (http://www.ncbi.nlm.nih.gov/gds/*).* After downloading raw microarray data, normalization and logarithmic method were employed to preprocess the data. Differential expression analysis of circRNAs was conducted using the Limma package, with the false discovery rate (FDR) threshold set at less than 0.05 for statistical significance.

### Weighted gene co-expression network analysis

A system biology method called WGCNA [15] was performed to find the modules of highly correlated genes and to relate these modules to traits. Firstly, the top 5000 median absolute deviation (MAD) circRNAs were selected to construct a network with biological significance, and the hclust function was used to cluster samples and to check the outliers. Secondly, the Pearson correlation was used to construct the correlation matrix, and the soft threshold power (*β*) was calculated to raise the correlation matrix to weighted adjacency matrix which was subsequently converted to a topological overlap matrix (TOM) and a dissimilarity TOM (dissTOM). Thirdly, modules were delineated through a one-step network construction process with a minimum module size of 30, and similar modules were merged with a threshold of 0.25. Finally, correlation analysis was used to assess the relationship between modules and CAD phenotypes. The CAD-related modules with a *P* value < 0.05 were selected for further analysis.

### Hub circRNAs identification

The VENNY 2.1 tool (https://bioinfogp.cnb.csic.es/tools/venny/index.html) was employed to identify overlapping circRNAs among DEcircRNAs and the circRNAs present in the CAD-related co-expression modules. LASSO regression analysis, incorporating penalty parameters and leave-one-out cross-validation, was applied to ascertain hub circRNAs from the overlapping set. LASSO regression shrinks the coefficient towards zero, with the degree of shrinkage dependent on a parameter λ under the minimum criteria.

### Quantitative real-time polymerase chain reaction (qRT-PCR)

Total RNA was extracted from the blood samples using the TRIzol™ kit (Bioteke, Beijing, CHN) according to the manufacturer’s instructions. The concentration and purity of total RNA were quantified by Multiskan GO Microplate Reader (ThermoFisher, Waltham, MA, USA). The GoScript™ RT Reagent Kit (Promega, Madison, WI, USA) was utilized to prepare the reverse transcription mixture system. The expression levels of circRNAs were quantified by qRT-PCR using SYBR Green GoTaq Master Mix (Promega, Madison, WI, USA) on the LightCycler 480II Real-Time PCR System (Roche, Rotkreuz, Switzerland) in triplicate for each sample. The PCR cycling conditions were as follows: one cycle at 95℃ for 30s, amplified by 40 cycles of 95℃ for 5s, 58℃ for 30s, and 72℃ for 30s. In this study, only five circRNAs’ specific primers were designed, and the used primer sequences are listed in Supplementary Table 1. The relative expression levels of RNA was calculated using the 2^−ΔΔCt^ method, which was normalized to *GAPDH*.

### Functional enrichment analysis

The circBank [16] database was employed to predict the top five downstream target miRNAs of the circRNAs, while the miRDIP [17] database was used to predict the possible target mRNAs of these miRNAs with a very high confidence filter (score class 1%=very high). Subsequently, the circRNA-miRNA-mRNA regulatory network was constructed and visualised using Cytoscape. Kyoto Encyclopedia of Genes and Genomes (KEGG) pathway and Gene Ontology (GO) analyses were conducted using the database of DAVID. A *P*-value of less than 0.05 was considered as significant threshold.

### Statistical analysis

Statistical analysis was performed with SPSS 26.0 software (SPSS, Inc., Chicago, IL, USA) and R Project. Quantitative variables were expressed as mean (standard deviation) or median (interquartile range, IQR) according to the distribution of variable whether conformed to normal distribution. According to corresponding data types, Student’s *t* test, Mann-whitney *U* test and *χ*^*2*^ test were selected to analyze the differences between two groups. The relationship between CAD and circRNAs was evaluated using conditional logistic regression to adjust for relevant confounders, and the results were presented as odds ratios (*OR*) and 95% confidence intervals (*CI*). Receiver operating characteristic (ROC) curves were constructed to estimate the discrimination of circRNAs for CAD. A base clinical model of CAD was constructed using multivariable logistic regression, incorporating the following variables: hypertension, dyslipidemia, diabetes mellitus and smoking. In reclassification analyses, the indexes of integrated discrimination improvement (IDI) and continuous net reclassification improvement (NRI) were implemented to quantify the added value of circRNAs. Discrimination accuracy was compared between the base clinical model and the base clinical model with circRNAs using the DeLong test. A two-tailed *P* value < 0.05 was considered statistically significant.

## Results

### Clinical characteristics of participants

The demographic, laboratory and clinical parameters of the participants are detailed in Table 1. The average age for the CAD and control groups was 60.45 ± 11.05 and 60.11 ± 11.43 years, respectively. Participants with CAD exhibited higher proportions of smoking history, diabetes mellitus, and hypertension compared to controls (*P* < 0.05). The fasting blood glucose, triglyceride, alanine aminotransferase (ALT) and hypersensitive C-reactive protein (hs-CRP) levels in the CAD group were significantly higher than in the control group (*P* < 0.05). The levels of high-density lipoprotein (HDL) and low-density lipoprotein (LDL) were significantly lower than in the control group (*P* < 0.05).

**Table 1:** Characteristics of the participants

Variables	Control (*n* = 100)	CAD (*n* = 100)	χ^2^/ t/ Z	*P*
**Demographic**				
Male (%)	57 (57.0)	57 (57.0)		-
Age, years	60.11 ± 11.43	60.45 ± 11.05	-0.214	0.831
Drinking history, %	25 (25.0)	27 (27.0)	0.104	0.747
Smoking history, %	37 (37.0)	52 (52.0)	4.555	**0.033**
BMI, kg/m^2^	24.33 ± 2.76	25.05 ± 3.54	-1.613	0.108
Diabetes mellitus, %	13 (13.0)	31 (31.0)	9.441	**0.002**
Hypertension, %	59 (59.0)	79 (79.0)	9.350	**0.002**
Dyslipidemia, %	46 (46.0)	72 (72.0)	13.973	**< 0.001**
**laboratory parameters**				
Fasting blood glucose (mmol/L)	5.30 (4.86, 5.74)	5.50 (5.02, 6.57)	-2.414	**0.016**
Triglyceride (mmol/L)	1.24 (0.89, 1.72)	1.53 (1.08, 1.77)	-2.097	**0.036**
Total Cholesterol (mmol/L)	4.63 ± 1.01	4.45 ± 1.04	1.242	0.216
HDL (mmol/L)	1.30 ± 0.35	1.19 ± 0.30	2.411	**0.017**
LDL (mmol/L)	2.54 ± 0.72	2.33 ± 0.73	2.123	**0.035**
ApoA1 (g/L)	1.11 ± 0.23	1.05 ± 0.27	1.627	0.105
ApoB (g/L)	0.73 ± 0.21	0.68 ± 0.19	1.562	0.120
Creatinine (µmol/L)	65.10 (56.03, 79.68)	71.40 (60.45, 83.20)	-1.863	0.062
ALT (U/L)	19.00 (13.00, 28.00)	23.00 (16.00, 35.00)	-2.441	**0.015**
AST (U/L)	22.00 (18.00, 27.00)	23.00 (20.00, 31.00)	-1.719	0.086
hs-CRP (mg/L)	0.85 (0.40, 1.89)	2.35 (0.70, 3.83)	-4.425	**< 0.001**
**Medication**				
Statin (%)	22 (22.0)	61 (61.0)	31.325	**< 0.001**
ACEI or ARB (%)	26 (26.0)	52 (52.0)	14.208	**< 0.001**
Diuretics (%)	17 (17.0)	18 (18.0)	0.035	0.852
CCB (%)	26 (26.0)	37 (37.0)	2.804	0.094
Beta-blocker (%)	20 (20.0)	32 (32.0)	3.742	0.053
Antiplatelet drug (%)	21 (21.0)	49 (49.0)	17.231	**< 0.001**

Abbreviations: BMI, Body Mass Index; HDL, high density lipoprotein; LDL, low density lipoprotein; ALT, Alanine aminotransferase; AST, Aspartate aminotransferase; hs-CRP, hypersensitive C-reactive protein; ACEI or ARB, angiotensin-converting enzyme inhibitor or angiotensin receptor blocker; CCB, Calcium-channel blocker

### Identification of CAD-related co-expression modules and circRNAs

**Fig. 1:**
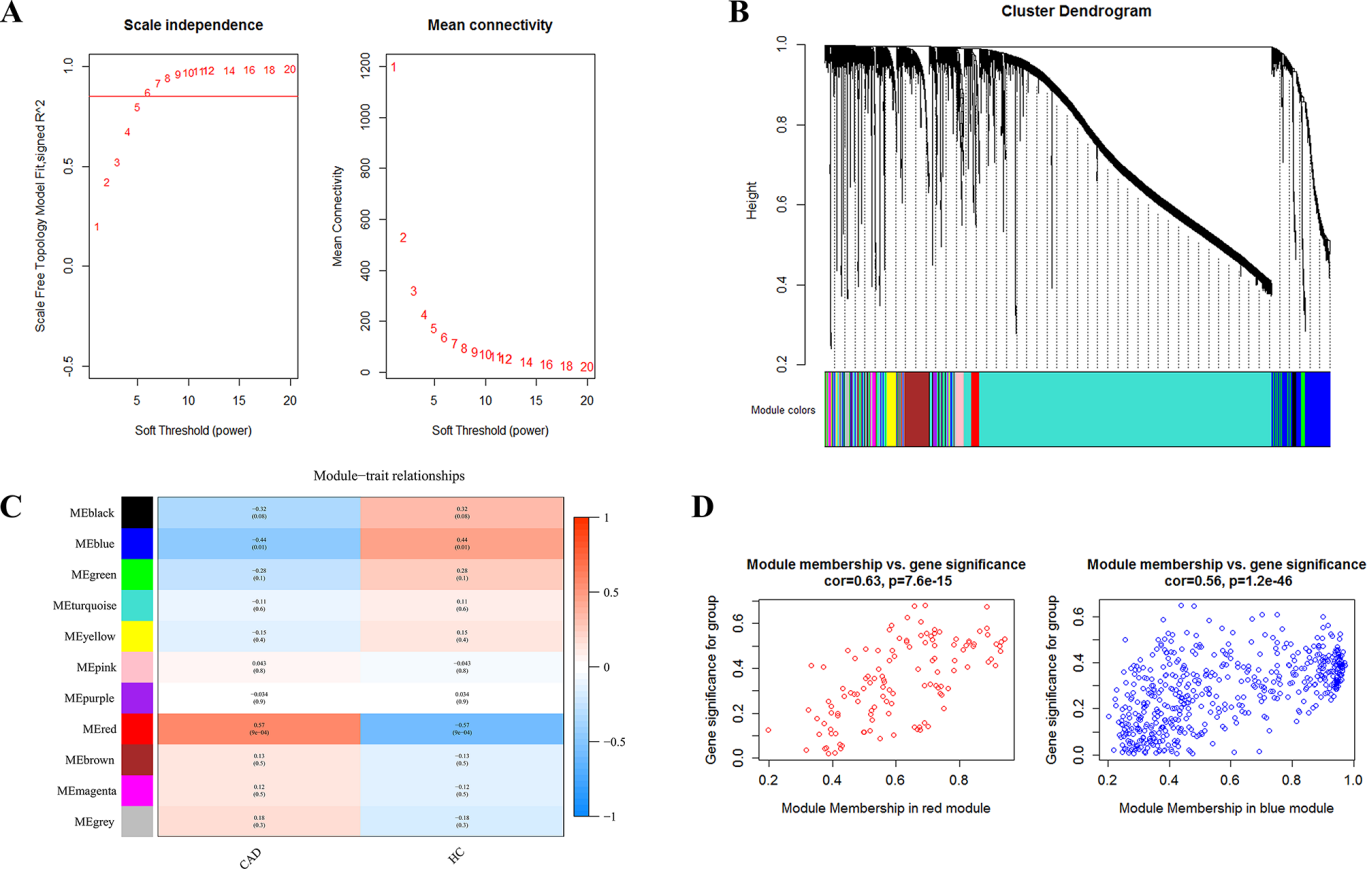
The WGCNA analysis of GSE115733. **(A)** Scale-free networks of scale independence and mean connectivity. **(B)** The cluster dendrogram of WGCNA. **(C)** A heatmap showing the correlation between each module eigengene and phenotype. **(D)** Scatter plots of gene significance (GS) vs. module membership (MM) in the red and blue modules. WGCNA, weighted gene co-expression network analysis; CAD, coronary artery disease

WGCNA was performed to identify the CAD-related co-expression modules and circRNAs. First, the optimal soft threshold power *β* was selected as 6 to meet the requirement of the scale-free topology index R^2^ exceeded 0.85 (Fig. 1A). Following the detection of co-expression modules and the merging of similar modules, 11 gene modules were identified, with sizes ranging from 50 to 3250 circRNAs (Fig. 1B). The red module (*r* = 0.57, *P* = 9e − 4) and the blue module (*r*=-0.44, *P* = 0.01) were found to be significantly associated with CAD (Fig. 1C). The significance of these CAD-related circRNAs in the red and blue modules are shown in Fig. 1D.

### Identification of CAD related hub circRNAs

**Fig. 2:**
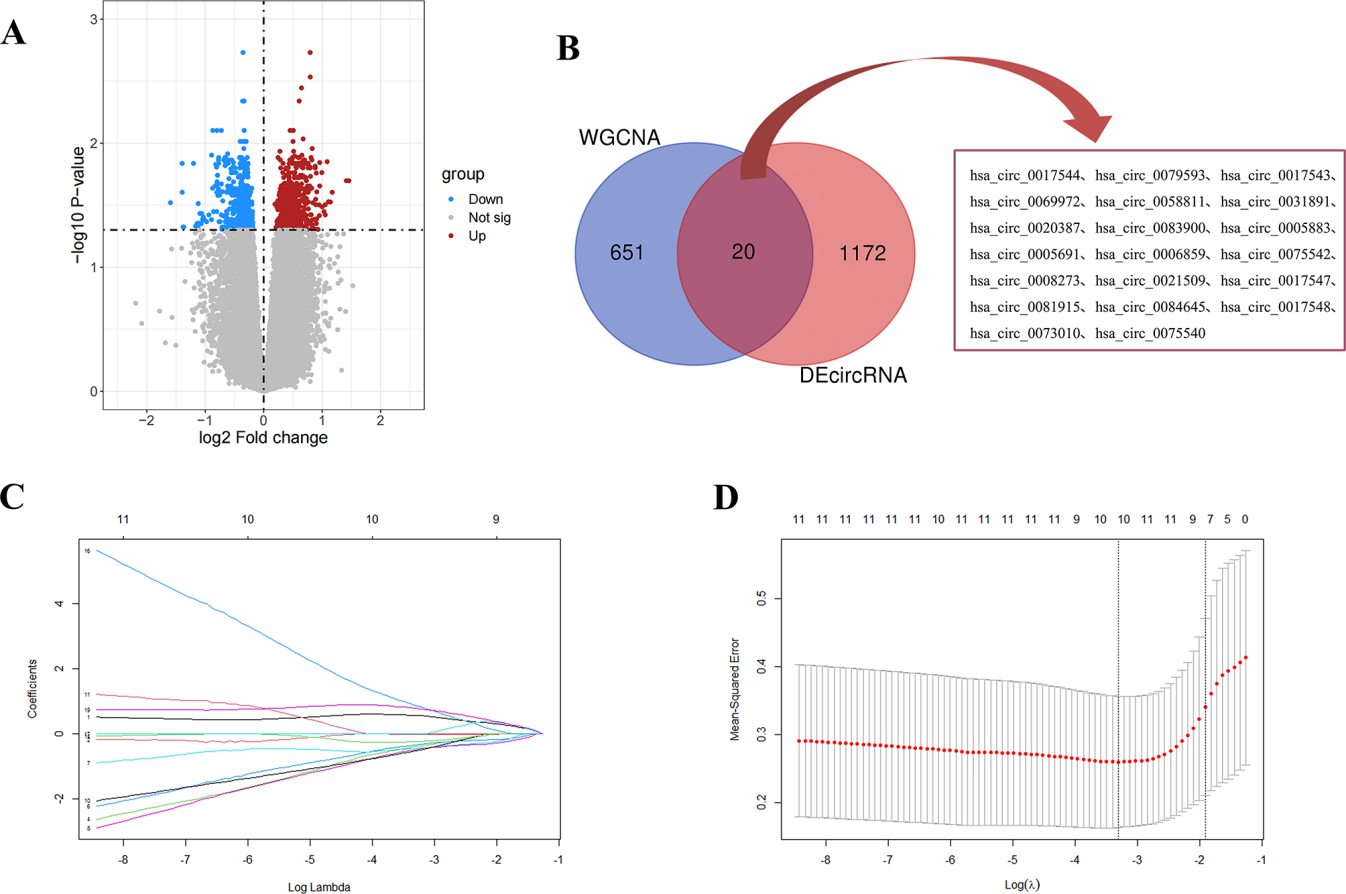
Identification of CAD related hub circRNAs. **(A)** Volcano showed expression of DEcircRNAs between the CADs and controls. **(B)** The veen plot showed the interaction between DEcircRNAs and circRNAs in red and blue modules. **(C)** LASSO coefficient profiles of the 10 circRNAs in CAD. **(D)** The log (lambda) sequence was used to construct a coefficient profile diagram. WGCNA, weighted gene co-expression network analysis; DEcircRNA, differentially expressed circRNA

In the differential analysis, we identified 599 up-regulated circRNAs and 593 down-regulated circRNAs (Fig. 2A). Subsequently, the 20 overlapping circRNAs among the DEcircRNAs and the circRNAs in the red and blue modules were selected (Fig. 2B). Moreover, LASSO regression analysis was employed to narrow down the 20 overlapping circRNAs, resulting in the identification of 10 hub circRNAs as potential diagnostic biomarkers for CAD (Fig. 2C, D). The details of these 10 hub circRNAs are presented in Supplementary Table 2.

### Identification of hsa_circ_0069972 and hsa_circ_0021509 as independent predictors of CAD

**Fig. 3:**
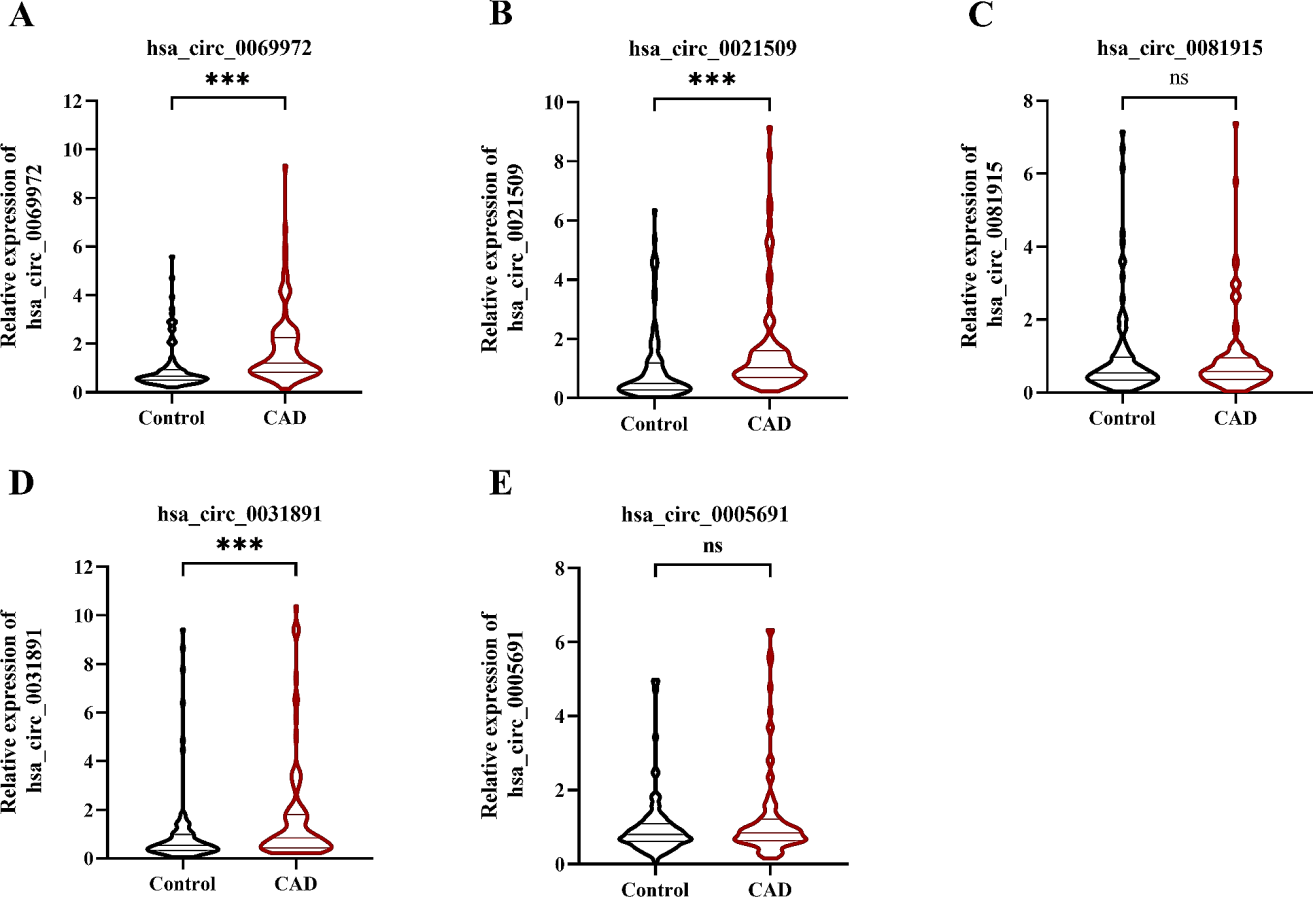
Circulating expression levels of five circular RNAs of CAD patients and controls. Quantitative real time polymerase chain reaction analysis of the circulating expression levels of **(A)** hsa_circ_0069972, **(B)** hsa_circ_0021509, **(C)** hsa_circ_0081915, **(D)** hsa_circ_0031891, and **(E)** hsa_circ_0005691 in the controls (*n* = 100) and CAD patients (*n* = 100). Data represent the median ± interquartile range, Mann-Whitney *U* test. CAD, coronary artery disease; ***, *P* < 0.001; ns, no significance

The expression levels of the five circRNAs were validated using qRT-PCR in 100 CAD patients and 100 controls. The expression levels of three circRNAs (hsa_circ_0069972, hsa_circ_0021509, and hsa_circ_0031891) were significantly higher in CAD patients that in controls (*P* < 0.05, Fig. 3A, B and D), Furthermore, the differential expression directionality was consistent with the result of the circRNA microarray (Supplementary Table 2). In addition, no significant difference was observed in the expression levels of hsa_circ_0081519 or hsa_circ_0005691 between the two groups (*P* > 0.05, Fig. 3C and E). The expression levels of circRNAs in CAD groups with or without relevant drugs were compared (Supplementary Fig. 1). Notably, the expression of hsa_circ_0031891 was significantly higher in CAD patients taking statins compared to those not taking statins (*P* < 0.05). However, no significant differences in expression levels of the three circRNAs were observed between the groups taking or not taking ACEI or ARB, CCBs, diuretics, beta-blockers and antiplatelet drugs (*P* > 0.05).

Conditional logistic regression was performed to assess the association between CAD and circRNAs. As presented in Table 2, the univariate logistic regression analysis showed a statistically significant relationship between circRNA expression levels and CAD. In the multivariable analysis, variables that differed between the CAD and control groups were adjusted as potential confounding factors. The peripheral blood levels of hsa_circ_0069972 (*OR* = 2.225, 95% *CI*:1.299–3.810, *P* = 0.004) and hsa_circ_0021509 (*OR* = 1.706, 95% *CI*: 1.111–2.620, *P* = 0.015) remained significantly associated with CAD after adjusting for the impact of smoking history, hypertension, diabetes mellitus, dyslipidemia, stains use, angiotensin-converting enzyme inhibitor or angiotensin receptor blocker use, and antiplatelet therapy. These results suggest that hsa_circ_0069972 and hsa_circ_0021509 are independent predictors of CAD.

**Table 2:** Logistic regression analysis of circRNAs for CAD

CircRNA	Univariable analysis		Multivariable analysis	
	OR (95% CI)	*P*	OR^a^ (95% CI)	*P*
hsa_circ_0069972	2.012 (1.404–2.883)	**< 0.001**	2.225 (1.299–3.810)	**0.004**
hsa_circ_0021509	1.632 (1.165–2.286)	**0.004**	1.706 (1.111–2.620)	**0.015**
hsa_circ_0031891	1.237 (1.028–1.487)	**0.024**	1.169 (0.949–1.442)	0.143

Note: a represents adjustment for smoking history, hypertension, diabetes mellitus, dyslipidemia, stains use, angiotensin-converting enzyme inhibitor or angiotensin receptor blocker use and antiplatelet therapy

### Performance of circRNAs as biomarkers of CAD

**Fig. 4:**
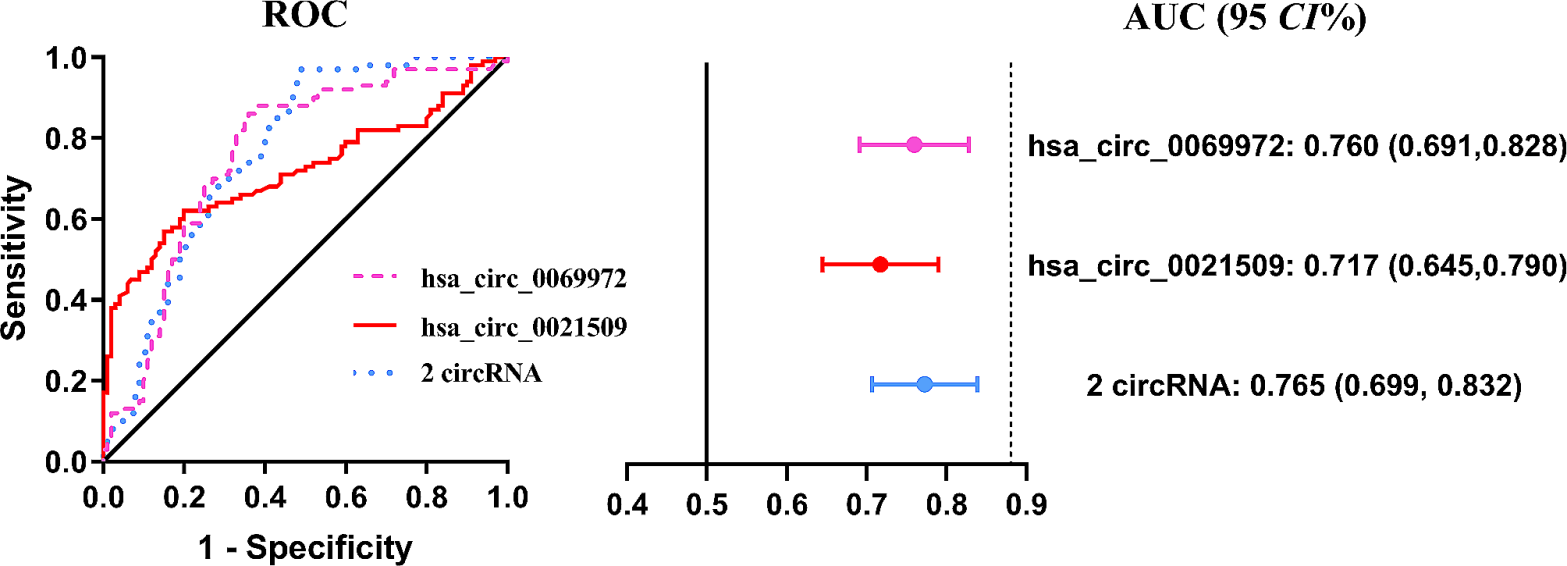
The ROC curve and AUC of circRNAs expression. ROC, receiver operating characteristic; AUC, area under the curve; *CI*: confidence interval

The diagnostic potential of hsa_circ_0069972 and hsa_circ_0021509 was evaluated using ROC curve analysis. The AUC for hsa_circ_0069972 and hsa_circ_0021509 were 0.760 (95% *CI*: 0.691–0.828, *P* < 0.001) and 0.717 (95% *CI*: 0.645–0.812, *P* < 0.001), respectively (Fig. 4). When combined, these two circRNAs yielded an AUC of 0.765 (95% *CI*: 0.699–0.832, *P* < 0.001); however this increase was not statistically significant when compared to the AUC for hsa_circ_0069972 alone (*P* > 0.05). The details of AUC, cut-off value, specificity, and sensitivity of each circRNA are presented in Supplementary Table 3.

To further explore the potential role of circRNAs as biomarkers for CAD, we evaluated the impact of incorporating hsa_circ_0069972 and hsa_circ_0021509 into a base clinical model that includes the following common cardiovascular risk factors: hypertension, dyslipidemia, diabetes mellitus and smoking history (Table 3). We found that the classification of patients was enhanced with the addition of circRNAs into the clinical model composed of conventional cardiovascular risk factors. The IDI and NRI of hsa_circ_0069972 were 0.131 and 0.170, respectively. And the IDI and NRI of hsa_circ_0021509 were 0.111 and 0.150, respectively.

**Table 3:** Performance of circular RNAs combined with clinical model for the diagnosis of CAD

Model	Discrimination		Reclassification				
	AUC (95% CI)	*P* (vs. CM)	IDI	*P* (vs. CM)	NRI	*P* (vs. CM)	
Clinical Model (CM)	0.708 (0.637–0.780)	NA	Reference model	NA	Reference model	NA	
CM + hs-CRP	0.736 (0.667–0.805)	**0.015**	0.094	**0.039**	0.100	**0.002**	
CM + hsa_circ_0069972	0.776 (0.711–0.840)	**0.003**	0.131	**0.030**	0.170	**0.004**	
CM + hsa_circ_0021509	0.744 (0.676–0.812)	**0.049**	0.111	**0.044**	0.150	**0.003**	
CM + 2 circRNA	0.786 (0.722–0.849)*	**0.001**	0.154	**0.010**	0.170	**0.002**	

Note: Clinical Model (CM) includes hypertension, dyslipidemia, diabetes mellitus and smoking history; * represents no significant difference between the AUC of CM + 2 circRNA and CM + hsa_circ_0069972 (*P* > 0.05). Abbreviations: CAD, coronary artery disease; NA, Not Applicable; AUC, area under the curve; CI, confidence interval; IDI, integrated discrimination improvement; NRI, net reclassification improvement

### Functional enrichment analysis

**Fig. 5:**
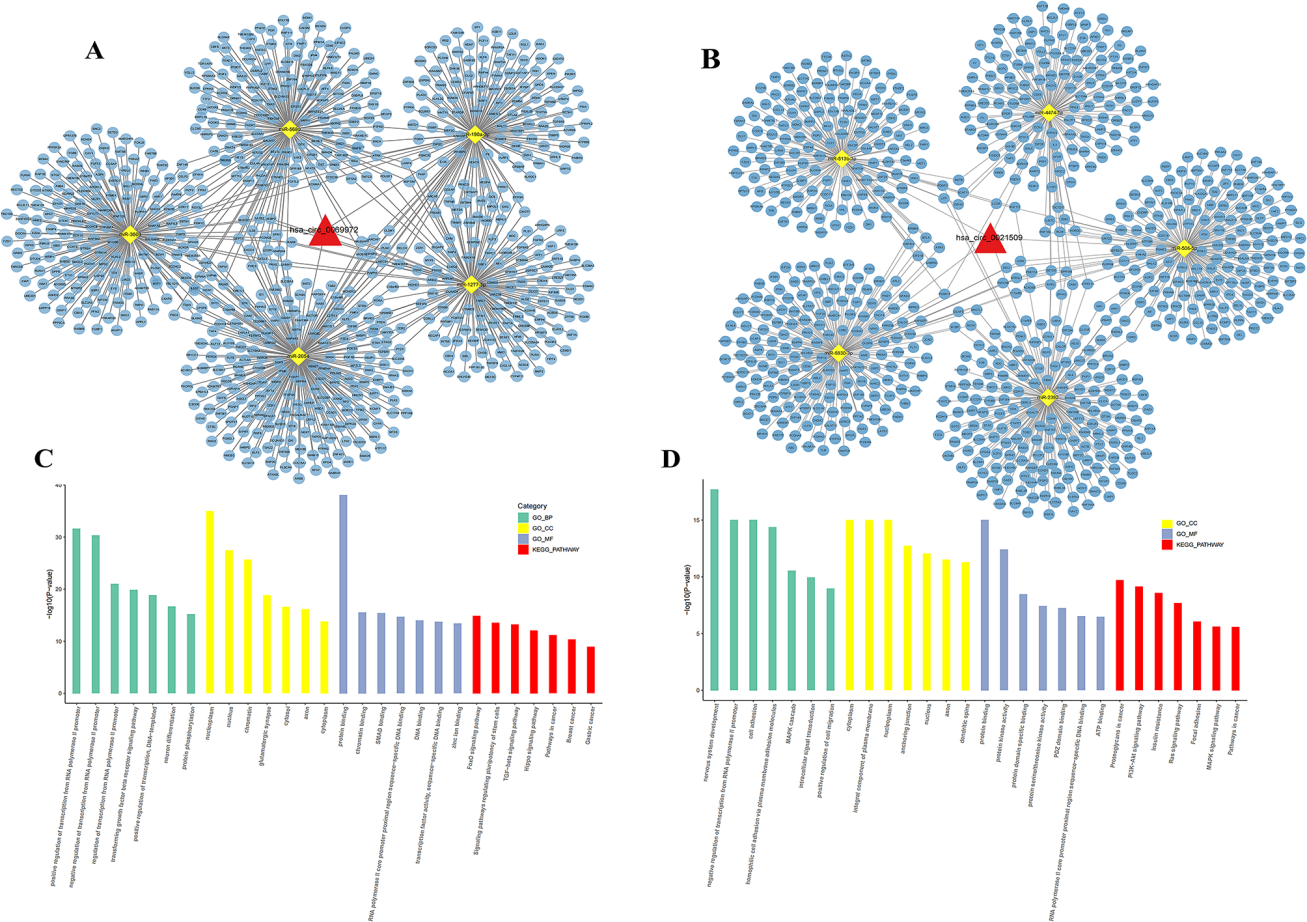
The circRNA-miRNA-mRNA regulatory network and functional enrichment analysis. **(A)** The hsa_circ_0069972-miRNA-mRNA regulatory network. The red triangle, yellow rhomboids and blue circles represent circRNAs, miRNAs and mRNAs, respectively. **(B)** The hsa_circ_0021509-miRNA-mRNA regulatory network. **(C)** Functional enrichment analysis of hsa_circ_0069972-miRNA-mRNA regulatory network. **(D)** Functional enrichment analysis of hsa_circ_0021509-miRNA-mRNA regulatory network. The *P*-value of terms was transformed to -log10(*P*-value)

The circRNA-miRNA-mRNA regulatory networks were constructed to elucidate the potential molecular mechanism of circRNAs. According to the circRNA-miRNA and miRNA-mRNA prediction, we constructed the hsa_circ_0069972-miRNA-mRNA network, composed of hsa_circ_0069972, 5 miRNA and 733 mRNA (Fig. 5A). In the same way, the hsa_circ_0021509-miRNA-mRNA network, composed of hsa_circ_0021509, 5 miRNA and 776 mRNA, was established as shown in Fig. 5B.

Then, functional enrichment analysis was performed on these two networks. The GO and KEGG analyses reshowed that hsa_circ_0069972-miRNA-mRNA network was enriched in *TGF-β* signaling pathway, *FoxO* signaling pathway, *Hippo* signaling pathway and so on (Fig. 5C), and hsa_circ_0021509-miRNA-mRNA network was enriched in cell adhesion, *PI3K/Akt* signaling pathway, *MAPK* signaling pathway, Ras signaling pathway and so on (Fig. 5D). The top seven enriched terms were shown in Supplementary Table 4.

## Discussion

As a multifactorial disease with high morbidity and mortality, CAD imposes a substantial burden on social economies [18]. It is of great significance to discriminate individuals with CAD and predict the risk of CAD [19, 20]. However, there is no generally accepted or used circulating biomarker for the detection of CAD currently. Increasing evidence has demonstrated that circRNAs play critical roles in the initiation, development, and progression of cardiovascular diseases [21, 22]. As a result, the purpose of this study was to identify potential circulating biomarkers of CAD, thereby guiding management based on cardiovascular risk.

In this study, we performed integrated bioinformatics analysis on the public circRNA expression chip dataset GSE115733 to determine the 20 hub circRNAs that may play vital roles in the pathogenesis and diagnosis of CAD. Previously, several studies have identified some biomarkers of CAD based on gene expression profiles by differentially expressed genes (DEGs) analysis [23, 24]. Confounding factors will reduce both the sensitivity and the specificity of the DEGs as disease biomarkers. Therefore, co-expression analysis such as WGCNA would be much more reasonable for determining the hub genes as biomarkers [25]. A significant advantage of WGCNA is its ability to identify the modules of highly correlated genes and establish a link between gene expression changes and disease characteristics. For instance, Wang *et a*l. utilized WGCNA to construct eight modules positively correlated with hepatocellular carcinoma samples, and identified has_circ_0000517 a*s* a potent biomarker for prognosis [26]. Jiang et al. have identified bladder tumor associated macrophages (TAM)-related modules using WGCNA and selected six key genes as potential biomarkers using cox regression analysis in combination with LASSO analysis [27]. To our knowledge, this is the first study to apply WGNCA to identify potential diagnostic circRNAs for CAD.

Accumulating evidence has indicated that circRNAs have great potential as diagnostic or prognostic biomarkers for diseases such as acute ischemic stroke [28], dilated cardiomyopathy [29], pancreatic ductal adenocarcinoma [30]. High stability, abundance and evolutionary conservation among species underline some of their important traits [31]. Zhou et al. performed a case-controlled study and confirmed that BTBD7_hsa_circ_0000563 can act as an independent predictor for CAD with the AUC of 0.690 [32]. Additionally, circRNA ZNF609 was identified to be lowly expressed in plasma of CAD patients, and it was inversely associated with the risks of CAD [33]. In this study, a case-controlled study was performed to explore the circulating expression of circRNAs and the role of circRNAs as diagnostic biomarkers of CAD. We observed increased circulating levels of hsa_circ_0069972 and hsa_circ_0021509 expression in CAD patients, even after adjusting for relevant confounders. This suggests that they may serve as independent predictors of CAD. In the ROC analysis, hsa_circ_0069972 and hsa_circ_0021509 had modest discriminative power. In line with this, a detailed analysis indicated that hsa_circ_0069972 and hsa_circ_0021509 correctly reclassified the patients misclassified by a multiparameter model of stable CAD exclusively based on the clinical history and cardiovascular risk factors. Our results were comparable to the previous study of Vilades et al., plasma hsa_circ_0001445 provided additional information beyond conventional risk factors in terms of reclassification of stable CAD patients into appropriate diagnostic groups [5]. The above results suggested that these two circRNAs could be used as potential non-invasive biomarkers for diagnosis of CAD.

Hsa_circ_0069972 is located at chr4:74861358–74,863,372, and originates from the gene *CXCL5*, which encodes CXC motif chemokine ligand 5. Previous research indicated that the plasma expression level of *CXCL5* was negatively correlated with the CAD severity score, suggesting *CXCL5* acts as a protective factor for CAD (*OR* = 0.46, 95%*CI*: 0.27–0.75) [34]. Another circRNA hsa_circ_0021509, located at chr11:22242642–22,279,300, is produced by the splicing of parental gene *ANO5*, which encodes anoctamin 5. The molecular mechanisms of these two circRNAs in CAD remain unreported.

CircRNAs are known to modulate mRNA expression by competing with miRNAs [35]. For instance, Cdr1as has been proposed to function as a sponge for miR-7 by reducing the number of freely available miR-7 molecules [36]. Zhong et al. found that circ_TET3 targeted *TET3* and *PPM1B* via sponging miR-361-3p, thereby mediating the proliferation and apoptosis of vascular smooth muscle cells (VSMC) in CAD [37]. In order to understand the potential role of these two circRNAs, we predicted the circRNA-miRNA-mRNA network and performed a functional enrichment analysis, respectively. As a result, hsa_circ_0069972-miRNA-mRNA network was enriched in *TGF-β*、*FoxO* and *Hippo* signaling pathways. *TGF-β* signaling pathway plays an important role in the development of CAD, such as stimulating chemotaxis of macrophages and fibroblasts, as well as increasing extracellular matrix synthesis [38, 39]. Ye et al. found that circ*COL1A1* could act as a sponge of miR-30a-5p to inhibit the expression of its target gene *SMAD1*, thereby inhibiting *TGF-β* signaling and promoting the phenotype switch of VSMC [40]. The *FoxO 1* is a crucial regulator of cell metabolism in heart tissues, where it is involved in cardiac regulation of glucose and lipid metabolic pathways, and endothelium, affecting cardiovascular pathophysiology [41]. Previous research found that inhibiting *YAP/TAZ*, effectors of the Hippo pathway, can suppress inflammation and reduce monocyte attachment, thereby retarding atherogenesis [42]. Functional enrichment revealed that the hsa_circ_0021509-miRNA-mRNA network is enriched in *PI3K/Akt* and *MAPK* signaling pathways. The *PI3K/Akt* pathway plays a crucial role in the survival, proliferation, and migration of macrophages, which may impact the development of atherosclerosis [43]. Jin et al. found that miRNA-26a-5p influences the proliferative and apoptotic abilities of endothelial cells isolated from CAD mice by targeting *PTEN* to activate *PI3K/AKT* pathway [44]. *MAPK* signaling pathway is also important for the progression of CAD, it can modulate the inflammatory response of atherosclerosis. Overall, hsa_circ_0069972 and hsa_circ_0021509 may participate in the development of CAD through the above pathways [45].

There are some limitations in this study that merit consideration. Firstly, due to the limitation of current method, the influence of confounders cannot be ruled out, and the association between circRNAs and CAD must be carefully evaluated. Secondly, the controls selected in this study were patients with coronary artery stenosis less than 50%. Therefore, the results of this study may have certain limitations when extrapolated to the general population. Finally, the potential pathways of circRNAs required confirmation by further laboratory studies.

Due to the increasing morbidity and mortality of CAD, it is imperative that develop novel non-invasive diagnostic and prognostic biomarkers. Studies have demonstrated that inflammatory biomarkers, like the C-reactive protein to albumin ratio (CAR), can be employed to monitor thrombotic status after the coronary angiography and offer more intensive treatment, thereby significantly reducing major adverse cardiac events (MACE) [46]. CircRNAs also have great potential in this regard, as these molecules exhibit excellent stability and conservation, they may reduce the number of unnecessary coronary angiography (CAG) procedures and allow for appropriate treatment planning [47]. Nevertheless, our comprehension of circRNAs remians limited, making the application of circRNAs in clinical practice exceedingly challenging. Despite the availability of these novel biomarkers, treatment should not be blindly changed. They should be used as auxiliary diagnostic tools, combined with other clinical information, to develop a more precise treatment plan for patients. Further in-depth studies are required to better understand the biology of circRNAs before these new diagnostic and therapeutic regimens can be routinely implemented. In general, hsa_circ_0069972 and hsa_circ_0021509 are independent predictors of CAD, showing modest discriminative power for CAD patients.

## Conclusion

In conclusion, hsa_circ_0069972 and hsa_circ_0021509 are identified by integrated analysis, and they are highly expressed in CAD patients. They may serve as novel biomarkers for CAD.

### Electronic supplementary material

Below is the link to the electronic supplementary material.

Supplementary Material 1

Supplementary Material 2

## Data Availability

CircRNAs expression profile data that support the findings of this study can been found in the Gene Expression Omnibus database with the primary accession code GSE115733.

## Abbreviations

ALT: Alanine aminotransferase
ARB: Angiotensin receptor blocker
ACEI: Angiotensin-converting enzyme inhibitor
AUC: Area under the curve
AST: Aspartate aminotransferase
BMI: Body mass index
CCB: Calcium-channel blocker
circRNA: Circular RNA
CI: Confidence intervals
CAD: Coronary artery disease
GO: Gene ontology
HDL: High density lipoprotein
hs-CRP: Hypersensitive C-reactive protein
IDI: Integrated discrimination improvement
KEGG: Kyoto Encyclopedia of Genes and Genomes
LASSO: Least absolute shrinkage and selection operator
LDL: Low density lipoprotein
MAD: Median absolute deviation
miRNA: MiroRNA
NRI: Net reclassification improvement
OR: Odds ratios
ROC: Receiver operating characteristic
TOM: Topological overlap matrix
VSMC: Vascular smooth muscle cells
WGCNA: Weighted gene co-expression network analysis
