# Supplementary material for: Identification and verification of circRNA biomarkers for coronary artery disease based on WGCNA and the LASSO algorithm
Source: BMC Cardiovasc Disord. 2024 Jun 17;24:305. doi: 10.1186/s12872-024-03972-2 (PMC11181640; doi:10.1186/s12872-024-03972-2)
Supplement: Supplementary file 1 — Supplementary Material 1 [file 12872_2024_3972_MOESM1_ESM.docx]

# SUPPLEMENTAL MATERIAL

**Identification of circRNA biomarker for coronary artery disease**

**through integrated analysis**

# Supplementary Material

**Supplementary Figure 1.** The expression levels of circRNAs in CAD groups with or without relevant drugs using.

**Supplementary Table 1.** Primer sequences for qRT-PCR.

**Supplementary Table 2.** The details of 10 hub circRNAs.

**Supplementary Table 3.** ROC analysis of each circRNA for assessing the discriminative accuracy.


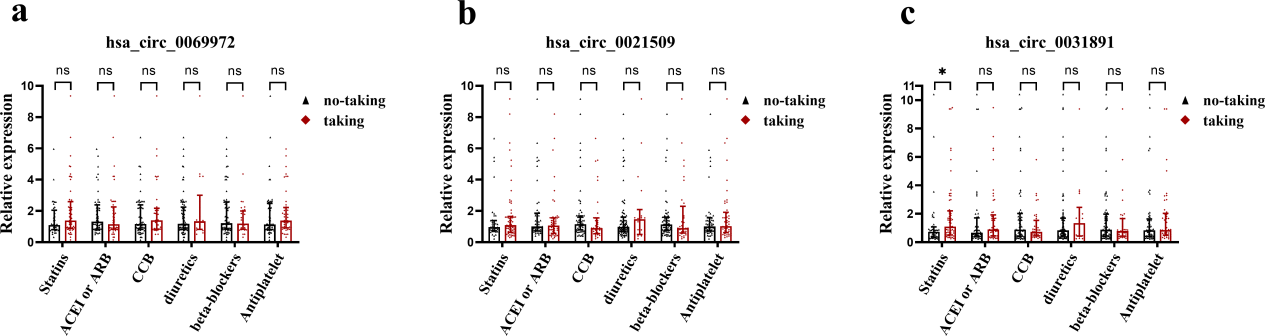


**Supplementary Figure 1.** The expression levels of circRNAs in CAD groups with or without relevant drugs using. *, *P*<0.05; ns, no significance.

| **Supplementary Table 1. Primer sequences for qRT-PCR.** | | |
| --- | --- | --- |
| Primer | | Primer sequence (5' - 3') |
| 1 | hsa_circ_0021509-F | TTCAGGAGCCACAGTGACATT |
|  | hsa_circ_0021509-R | TTGCCTAATCCCATCTCGGAA |
| 2 | hsa_circ_0031891-F | GCCCGGCTACATGAATAACA |
|  | hsa_circ_0031891-R | CAATCCAAGAGTCTCTGAGGTTA |
| 3 | hsa_circ_0005691-F | TCTTTCAGAAGGGTGGTCCAT |
|  | hsa_circ_0005691-R | TCATAAAGTTATAACCAACATCAGGC |
| 4 | hsa_circ_0081915-F | GCCGAAATGCTTGTGAATGC |
|  | hsa_circ_0081915-R | GTTGGGATCACACACGGAAC |
| 5 | hsa_circ_0069972-F | ACTTCATGGAAACAAGGAAAAC |
|  | hsa_circ_0069972-R | TGGGTTCAGAGACCTCCAGA |
| 6 | GAPDH-F | CCCACATGGCCTCCAAGGAGTA |
|  | GAPDH-R | GTGTACATGGCAACTGTGAGGAGG |
| Abbreviations: F, forward; R, reverse. | | |

| **Supplementary Table 2. The details of 10 hub circRNAs.** | | | | |
| --- | --- | --- | --- | --- |
| circRNA ID | Location | Length（bp） | Parental gene | Fold change |
| hsa_circ_0017544 | chr 10: 5139625-5149878 | 903 | *AKR1C3* | -1.201 |
| hsa_circ_0079593 | chr 7: [23381682-23383472](http://genome.mdc-berlin.de/cgi-bin/hgTracks?db=hg19&position=chr7:23381682-23383472&hubUrl=http://bimsbstatic.mdc-berlin.de/hubs/rajewsky/circBase/hub.txt) | 262 | *[IGF2BP3](http://www.ncbi.nlm.nih.gov/gene?term=(IGF2BP3%5bPreferred)* | 1.109 |
| hsa_circ_0069972 | chr 4: [74861358-74863372](http://genome.mdc-berlin.de/cgi-bin/hgTracks?db=hg19&position=chr4:74861358-74863372&hubUrl=http://bimsbstatic.mdc-berlin.de/hubs/rajewsky/circBase/hub.txt) | 2014 | *CXCL5* | 1.032 |
| hsa_circ_0031891 | chr 14: [51387673-51401903](http://genome.mdc-berlin.de/cgi-bin/hgTracks?db=hg19&position=chr14:51387673-51401903&hubUrl=http://bimsbstatic.mdc-berlin.de/hubs/rajewsky/circBase/hub.txt) | 427 | *[PYGL](http://www.ncbi.nlm.nih.gov/gene/?term=5836)* | 1.458 |
| hsa_circ_0020387 | chr 10: [128768965-128823047](http://genome.mdc-berlin.de/cgi-bin/hgTracks?db=hg19&position=chr10:128768965-128823047&hubUrl=http://bimsbstatic.mdc-berlin.de/hubs/rajewsky/circBase/hub.txt) | 1442 | *[DOCK1](http://www.ncbi.nlm.nih.gov/gene?term=(DOCK1%5bPreferred)* | 1.141 |
| hsa_circ_0083900 | [chr 8](http://genome.mdc-berlin.de/cgi-bin/hgTracks?db=hg19&position=chr8:32472031-32585596&hubUrl=http://bimsbstatic.mdc-berlin.de/hubs/rajewsky/circBase/hub.txt): [32472031-32585596](http://genome.mdc-berlin.de/cgi-bin/hgTracks?db=hg19&position=chr8:32472031-32585596&hubUrl=http://bimsbstatic.mdc-berlin.de/hubs/rajewsky/circBase/hub.txt) | 232 | *[NRG1](http://www.ncbi.nlm.nih.gov/gene?term=(NRG1%5bPreferred)* | 1.414 |
| hsa_circ_0005691 | chr 10: [96267023-96271411](http://genome.mdc-berlin.de/cgi-bin/hgTracks?db=hg19&position=chr10:96267023-96271411&hubUrl=http://bimsbstatic.mdc-berlin.de/hubs/rajewsky/circBase/hub.txt) | 242 | *[TBC1D12](http://www.ncbi.nlm.nih.gov/gene/?term=23232)* | 0.900 |
| hsa_circ_0021509 | chr 11: [22242642-22279300](http://genome.mdc-berlin.de/cgi-bin/hgTracks?db=hg19&position=chr11:22242642-22279300&hubUrl=http://bimsbstatic.mdc-berlin.de/hubs/rajewsky/circBase/hub.txt) | 1227 | *[ANO5](http://www.ncbi.nlm.nih.gov/gene/?term=203859)* | 1.054 |
| hsa_circ_0081915 | chr 7: [107575859-107600279](http://genome.mdc-berlin.de/cgi-bin/hgTracks?db=hg19&position=chr7:107575859-107600279&hubUrl=http://bimsbstatic.mdc-berlin.de/hubs/rajewsky/circBase/hub.txt) | 1874 | *[LAMB1](http://www.ncbi.nlm.nih.gov/gene/?term=3912)* | -1.033 |
| hsa_circ_0073010 | [chr 5](http://genome.mdc-berlin.de/cgi-bin/hgTracks?db=hg19&position=chr5:73923233-73932323&hubUrl=http://bimsbstatic.mdc-berlin.de/hubs/rajewsky/circBase/hub.txt): [73923233-73932323](http://genome.mdc-berlin.de/cgi-bin/hgTracks?db=hg19&position=chr5:73923233-73932323&hubUrl=http://bimsbstatic.mdc-berlin.de/hubs/rajewsky/circBase/hub.txt) | 4399 | *[ENC1](http://www.ncbi.nlm.nih.gov/gene?term=(ENC1%5bPreferred)* | -1.395 |

| **Supplementary Table 3. ROC analysis of each circRNA for assessing the discriminative accuracy.** | | | | | | |
| --- | --- | --- | --- | --- | --- | --- |
| circRNA | AUC (95% *CI*) | *P* | sensitivity | specificity | Youden index | cut off |
| hsa_circ_0069972 | 0.760 (0.691-0.828) | <0.001 | 0.88 | 0.62 | 0.50 | 0.740 |
| hsa_circ_0021509 | 0.717 (0.645-0.812) | <0.001 | 0.80 | 0.62 | 0.42 | 0.652 |
| 2 circRNA | 0.765 (0.699-0.832) | <0.001 | 0.97 | 0.51 | 0.48 | 0.386 |
| Abbreviations: circRNA, circular RNA; AUC, area under the curve; *CI*, confidence interval. | | | | | | |

| **Supplementary Table 4. Functional enrichment analysis** | | | |
| --- | --- | --- | --- |
| **Identification** | **Term** | **Count** | ***p-*Value** |
| **hsa_circ_0069972** | | | |
| **GO-BP** |  |  |  |
| GO:0045944 | positive regulation of transcription from RNA polymerase II promoter | 98 | 6.58E-11 |
| GO:0000122 | negative regulation of transcription from RNA polymerase II promoter | 84 | 1.22E-10 |
| GO:0006357 | regulation of transcription from RNA polymerase II promoter | 111 | 8.97E-07 |
| GO:0007179 | transforming growth factor beta receptor signaling pathway | 20 | 2.12E-06 |
| GO:0045893 | positive regulation of transcription, DNA-templated | 59 | 4.82E-06 |
| GO:0030182 | neuron differentiation | 24 | 3.49E-05 |
| GO:0006468 | protein phosphorylation | 43 | 1.28E-04 |
| **GO-CC** |  |  |  |
| GO:0005654 | nucleoplasm | 224 | 3.72E-13 |
| GO:0005634 | nucleus | 291 | 3.41E-10 |
| GO:0000785 | chromatin | 82 | 1.36E-09 |
| GO:0098978 | glutamatergic synapse | 38 | 9.83E-07 |
| GO:0005829 | cytosol | 252 | 7.32E-06 |
| GO:0030424 | axon | 35 | 9.52E-06 |
| GO:0005737 | cytoplasm | 248 | 8.25E-05 |
| **GO-MF** |  |  |  |
| GO:0005515 | protein binding | 552 | 2.33E-14 |
| GO:0003682 | chromatin binding | 42 | 5.44E-05 |
| GO:0046332 | SMAD binding | 13 | 5.44E-05 |
| GO:0000978 | RNA polymerase II core promoter proximal region sequence-specific DNA binding | 79 | 8.49E-05 |
| GO:0003677 | DNA binding | 84 | 1.33E-04 |
| GO:0003700 | transcription factor activity, sequence-specific DNA binding | 45 | 1.45E-04 |
| GO:0008270 | zinc ion binding | 61 | 1.75E-04 |
| **KEGG** |  |  |  |
| hsa04068 | FoxO signaling pathway | 20 | 7.52E-05 |
| hsa04550 | Signaling pathways regulating pluripotency of stem cells | 20 | 1.30E-04 |
| hsa04350 | TGF-beta signaling pathway | 16 | 1.30E-04 |
| hsa04390 | Hippo signaling pathway | 20 | 3.07E-04 |
| hsa05200 | Pathways in cancer | 41 | 6.30E-04 |
| hsa05224 | Breast cancer | 18 | 0.00116626 |
| hsa05226 | Gastric cancer | 17 | 0.00417434 |
| **hsa_circ_0021509** | | | |
| **GO-BP** |  |  |  |
| GO:0007399 | nervous system development | 42 | 7.04E-05 |
| GO:0000122 | negative regulation of transcription from RNA polymerase II promoter | 71 | 2.38E-04 |
| GO:0007155 | cell adhesion | 48 | 2.47E-04 |
| GO:0007156 | homophilic cell adhesion via plasma membrane adhesion molecules | 23 | 4.99E-04 |
| GO:0000165 | MAPK cascade | 17 | 0.01816141 |
| GO:0035556 | intracellular signal transduction | 36 | 0.02760274 |
| GO:0030335 | positive regulation of cell migration | 24 | 0.06244957 |
| **GO-CC** |  |  |  |
| GO:0005737 | cytoplasm | 270 | 1.59E-05 |
| GO:0005887 | integral component of plasma membrane | 97 | 1.59E-05 |
| GO:0005654 | nucleoplasm | 203 | 2.23E-05 |
| GO:0070161 | anchoring junction | 40 | 4.48E-04 |
| GO:0005634 | nucleus | 274 | 7.02E-04 |
| GO:0030424 | axon | 32 | 9.98E-04 |
| GO:0043197 | dendritic spine | 20 | 0.00107795 |
| **GO-MF** |  |  |  |
| GO:0005515 | protein binding | 547 | 5.50E-05 |
| GO:0004672 | protein kinase activity | 35 | 0.00206650 |
| GO:0019904 | protein domain specific binding | 24 | 0.07066021 |
| GO:0004674 | protein serine/threonine kinase activity | 30 | 0.14190887 |
| GO:0030165 | PDZ domain binding | 12 | 0.14190887 |
| GO:0000978 | RNA polymerase II core promoter proximal region sequence-specific DNA binding | 68 | 0.20062108 |
| GO:0005524 | ATP binding | 83 | 0.20062108 |
| **KEGG** |  |  |  |
| hsa04068 | FoxO signaling pathway | 20 | 7.52E-05 |
| hsa04550 | Signaling pathways regulating pluripotency of stem cells | 20 | 1.30E-04 |
| hsa04350 | TGF-beta signaling pathway | 16 | 1.30E-04 |
| hsa04390 | Hippo signaling pathway | 20 | 3.07E-04 |
| hsa05200 | Pathways in cancer | 41 | 6.30E-04 |
| hsa05224 | Breast cancer | 18 | 0.00116626 |
| hsa05226 | Gastric cancer | 17 | 0.00417434 |
| Abbreviations: GO, gene ontology; BP, biological process; CC, cellular component; MF, molecular function; KEGG: Kyoto Encyclopedia of Genes and Genomes. | | | |
