# Supplementary figures and images for: Identification and verification of circRNA biomarkers for coronary artery disease based on WGCNA and the LASSO algorithm
Source: BMC Cardiovasc Disord. 2024 Jun 17;24:305. doi: 10.1186/s12872-024-03972-2 (PMC11181640; doi:10.1186/s12872-024-03972-2)

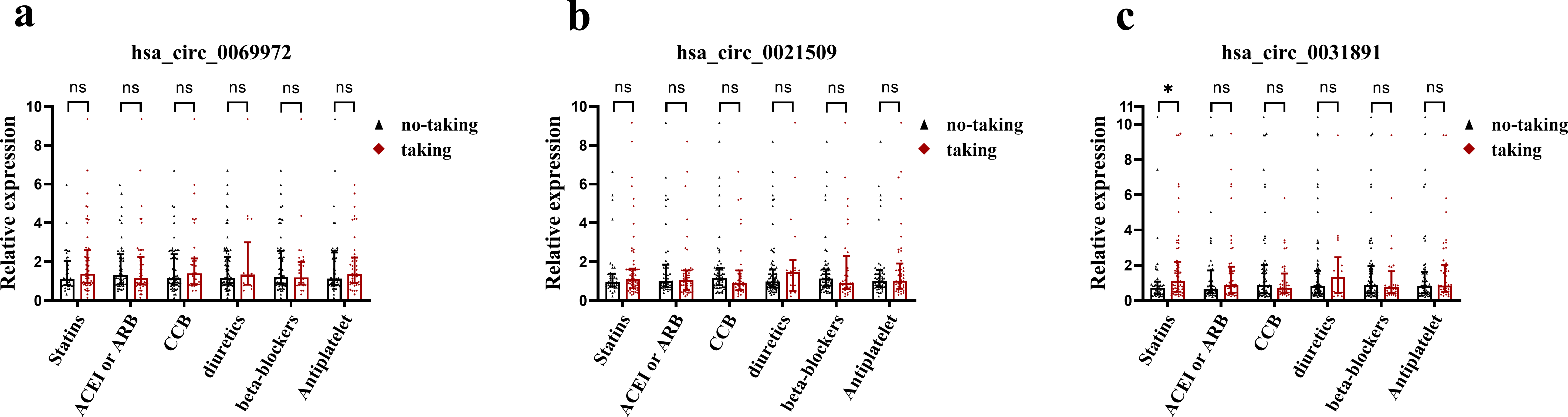

Supplement: Supplementary file 2 — Supplementary Material 2 [file 12872_2024_3972_MOESM2_ESM.png]
